# Analysis of mitochondrial function in human induced pluripotent stem cells from patients with mitochondrial diabetes due to the A3243G mutation

**DOI:** 10.1038/s41598-018-19264-7

**Published:** 2018-01-17

**Authors:** Masaki Matsubara, Hajime Kanda, Hiromi Imamura, Mayumi Inoue, Michio Noguchi, Kiminori Hosoda, Akira Kakizuka, Kazuwa Nakao

**Affiliations:** 1 Medical Innovation Center, Kyoto University Graduate School of Medicine, 53 Shogoin Kawahara-cho, Sakyo-ku, Kyoto 606-8507 Japan; 2 Department of Diabetes, Endocrinology and Nutrition, Kyoto University Graduate School of Medicine, 54 Shogoin Kawahara-cho, Sakyo-ku, Kyoto 606-8507 Japan; 3 Kyoto University Graduate School of Biostudies, Yoshida-Konoecho, Sakyo-ku, Kyoto 606-8501 Japan; 4 Department of Human Health Science, Kyoto University Graduate School of Medicine, 53 Shogoin Kawahara-cho, Sakyo-ku, Kyoto 606-8507 Japan

## Abstract

We previously established human induced pluripotent stem (iPS) cells in two diabetic patients from different families with the mitochondrial A3243G mutation and isolated isogenic iPS cell clones with either undetectable or high levels of the mutation in both patients. In the present study, we analyzed the mitochondrial functions of two mutation-undetectable and two mutation-high clones in each patient through four methods to assess complex I activity, mitochondrial membrane potential, mitochondrial respiration, and mitochondrial ATP production. In the first patient, complex I activity, mitochondrial respiration, and mitochondrial ATP production were decreased in the mutation-high clones compared with the mutation-undetectable clones, and mitochondrial membrane potential was decreased in a mutation-high clone compared with a mutation-undetectable clone. In the second patient, complex I activity was decreased in one mutation-high clone compared with the other clones. The other parameters showed no differences in any clones. In addition, the complex I activity and mitochondrial respiration of the mutation-undetectable clones from both patients were located in the range of those of iPS cells from healthy subjects. The present study suggests that the mitochondrial function of the mutation-undetectable iPS cell clones obtained from two patients with the A3243G mutation is comparable to the control iPS cells.

## Introduction

Like embryonic stem (ES) cells, human induced pluripotent stem (iPS) cells, which are generated from somatic cells, possess pluripotency in all three germ layers; therefore, they are considered promising sources for cell-replacement therapy and useful tools for developing disease models or drug screening^1,2^. Recently, iPS cell-derived retinal pigment epithelial cells were transplanted to a patient with neovascular age-related macular degeneration and no serious events were observed at two years of follow-up^3^. Although a major concern about cell replacement therapy is tumorigenicity^4^, integration-free methods to generate iPS cells^5^, *in vivo* tumorigenicity tests^6^, and precise assessments of genome integrity^3^ could prevent tumorigenesis. Mitochondria contain their own genomes, known as mitochondrial DNA (mtDNA), and mtDNA mutations induce mitochondrial dysfunction, thereby causing mitochondrial diseases. An A to G mutation at position 3243 (A3243G) in the mitochondrial tRNA^Leu(UUR)^ gene is one of the most frequent mtDNA mutations^7^. It is associated with various clinical symptoms, such as diabetes mellitus, hearing loss, and cardiomyopathy, and found in approximately 80% of patients with mitochondrial encephalopathy, lactic acidosis, and stroke-like episodes (MELAS)^8–11^.

Previously, we established human iPS cell lines in two patients from different families carrying the mtDNA A3243G mutation (Mt iPS cells): patient 1 (Pt1) was diabetic, and patient 2 (Pt2) had diabetes and MELAS^12^. A striking feature of Mt iPS cells is that mtDNA mutation frequencies decrease to undetectable levels in approximately half of the clones while they increase to more than 70% in the other half of the clones; this effect is observed in both patients. We speculate that two separate types of iPS cells, namely, mutation-undetectable clones (less than 2%) and mutation-high clones (more than 70%), arise through the process of iPS cell generation. Following our discovery, several studies have also reported that mutation-undetectable iPS cells can be generated in the process of reprograming, probably led by bimodal segregation toward homoplasmy, from patients carrying mtDNA mutations, thus suggesting a common mechanism for mitochondrial diseases^13–16^.

The mitochondrial function of the mutation-undetectable Mt iPS cell clones remains to be elucidated. It is unclear whether the mitochondrial function is different between the mutation-undetectable Mt iPS cell clones and the iPS cells from healthy subjects. In the present study, we analyzed the mitochondrial function of the Mt iPS cell clones, three control iPS cell lines from healthy subjects, and one ES cell line with regard to the following four parameters: complex I activity, mitochondrial membrane potential, oxygen consumption rate (OCR), and cytosolic ATP concentration.

## Results

### Complex I activity analyzed via colorimetric assay

**Figure 1:**
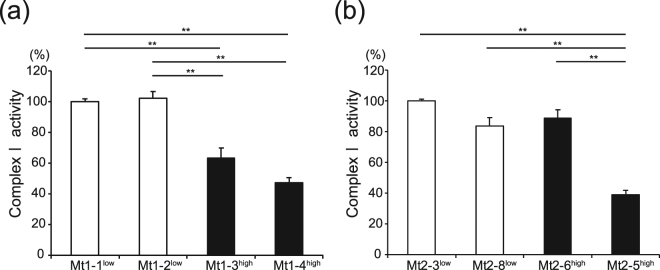
Complex I activity of the Mt iPS cell clones. The complex I activity of the Mt iPS cell clones was analyzed. The data of the Mt1 iPS cell clones (**a**) were normalized to Mt1-1^low^, and those of the Mt2 iPS cell clones (**b**) were normalized to Mt2-3^low^. The data are expressed as the means ± SE. *n* = 5–8. **P < 0.01, ANOVA with a post hoc test (Tukey’s test).

The complex I activity of iPS cells from Pt1 (Mt1 iPS cells) was decreased in the mutation-high clones, designated by a superscript ‘high’, compared with the mutation-undetectable clones, designated by a superscript ‘low’. The enzymatic activities of complex I were 102.2% (Mt1-2^low^), 63.4% (Mt1-3^high^), and 47.2% (Mt1-4^high^) of the activity of Mt1-1^low^ (calculated as a percentage of Mt1-1^low^; Fig. 1a, *n* = 5–8). The complex I activity of Mt1-3^high^ and Mt1-4^high^ was statistically significantly lower than that of Mt1-1^low^ and Mt1-2^low^. The complex I activity of iPS cells from Pt2 (Mt2 iPS cells) was decreased in Mt2-5^high^, which had the highest mutation frequency (85%), compared with the other clones. The complex I activities of the Mt2 iPS cell clones were 83.6% (Mt2-8^low^), 88.5% (Mt2-6^high^), and 38.9% (Mt2-5^high^) of that found in Mt2-3^low^ (Fig. 1b, *n* = 5–6). The complex I activity of Mt2-5^high^ was statistically significantly lower than that of Mt2-3^low^, Mt2-8^low^, and Mt2-6^high^. The complex I activity among Mt2-3^low^, Mt2-8^low^, and Mt2-6^high^ was not statistically significantly different. Moreover, the complex I activity of the three iPS cell lines (B7^1^, W12^17^, and TIG107^18^) generated from healthy subjects and the ES cell line (KhES-1^19^) were compared with that of the mutation-undetectable Mt iPS cell clones (Mt1-1^low^ and Mt2-3^low^) and the complex I activity was comparable between the mutation-undetectable Mt iPS cell clones and the control pluripotent stem cells (PSCs) (Supplementary Fig. 1, *n* = 10–15).

### Mitochondrial membrane potential analyzed with fluorescence-activated cell sorting (FACS)

**Figure 2:**
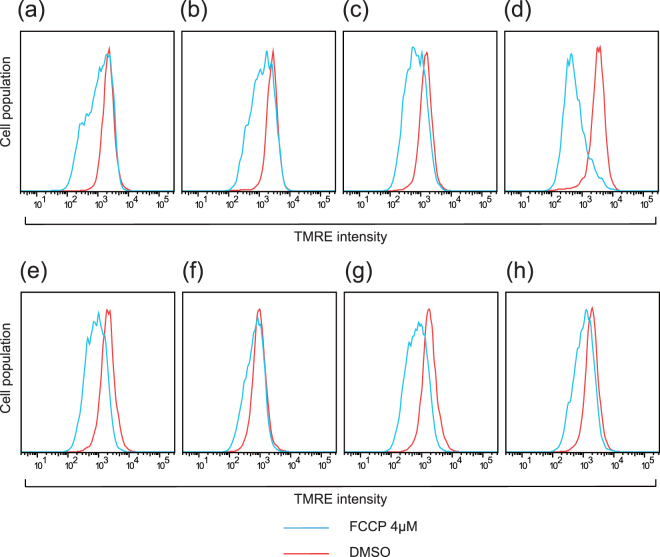
Mitochondrial membrane potential of the Mt iPS cell clones, as measured by FACS analysis. Representative histograms of TMRE intensity among the Mt1 iPS cell clones ((**a**): 1-1^low^, (**b**): 1-2^low^, (**c**): 1-3^high^, (**d**): 1-4^high^) and the Mt2 iPS cell clones ((**e**): 2-3^low^, (**f**): 2-8^low^, (**g**): 2-6^high^, (**h**): 2-5^high^) are shown. The Mt iPS cells were treated with 4 μM FCCP or DMSO for 10 min and analyzed independently. The TMRE peak decreased after FCCP administration was statistically significantly lower in Mt1-4^high^ than in Mt1-1^low^ among the Mt1 iPS cell clones, while it was not statistically significantly different among the Mt2 iPS cell clones assessed using ANOVA with a post hoc test (Tukey’s test).

MitoTracker Green and tetramethylrhodamine, ethyl ester, perchlorate (TMRE) intensity showed that in the basal state, the mutation-undetectable clones and the mutation-high clones did not show different peak positions among the Mt1 and Mt2 iPS cell clones (data not shown). The TMRE peak position of the Mt iPS cells shifted to the left after treatment with 4 μM carbonyl cyanide 4-trifluoromethoxy-phenylhydrazone (FCCP). Among the Mt1 iPS cell clones, the intensity of the TMRE peaks were decreased to 78.4% (Mt1-1^low^), 46.9% (Mt1-2^low^), 39.5% (Mt1-3^high^), and 16.6% (Mt1-4^high^) after FCCP administration shown in histograms with the horizontal axis as the fluorescence intensity and the vertical axis as the percentage of cells (Fig. 2a,b,c and d, *n* = 4). The TMRE peak after FCCP treatment was statistically significantly lower in Mt1-4^high^ than in Mt1-1^low^. However, the TMRE peaks were decreased to 34.6% (Mt2-3^low^), 65.9% (Mt2-8^low^), 26.4% (Mt2-6^high^), and 38.3% (Mt2-5^high^) after FCCP administration; the peak decrease was not statistically significantly different among the Mt2 iPS cell clones (Fig. 2e,f,g and h, *n* = 3).

### Oxygen consumption rate measured using an extracellular flux analyzer

OCR was measured using a series of mitochondria-specific inhibitors or a respiration uncoupling reagent to determine the bioenergetic profiles of the iPS cells. This sequence provides the following profiles of mitochondrial activity:(1) basal respiration;(2) the component of OCR used for ATP production, which is the difference between the basal OCR and the oligomycin A-repressed OCR;(3) the component of OCR representing proton leak across the mitochondrial inner membrane, which is the difference between the oligomycin A-repressed OCR and the rotenone plus antimycin A-inhibited OCR; and (4) the spare respiratory capacity, which is the difference between the FCCP-induced OCR and the basal OCR (Supplementary Fig. 2).

**Figure 3:**
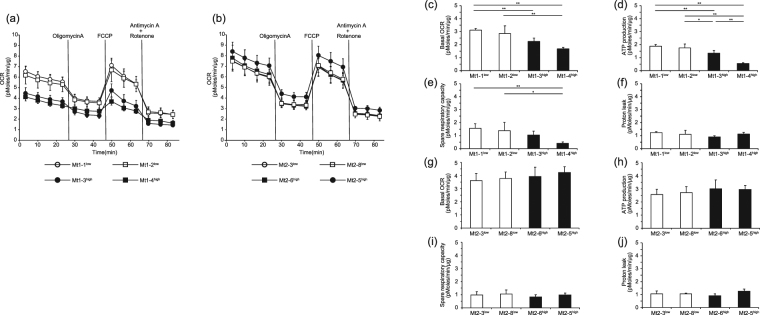
The oxygen consumption rate (OCR) of the Mt iPS cell clones measured using an XF96 Extracellular Flux Analyzer. Representative data for the Mt1 iPS cell clones are shown as follows: (**a**) time course (open circle, Mt1-1^low^; open square, Mt1-2^low^; closed circle, Mt1-3^high^; closed square, Mt1-4^high^), (**c**) basal OCR, (**d**) ATP production, (**e**) spare respiratory capacity, and (**f**) proton leak. Representative data for the Mt2 iPS cell clones are shown as follows: (**b**) time course (open circle, Mt2-3^low^; open square, Mt2-8^low^; closed square, Mt2-6^high^; closed circle, Mt2-5^high^), (**g**) basal OCR, (**h**) ATP production, (**i**) spare respiratory capacity, and (**j**) proton leak. OCR was normalized to total protein and is expressed as the mean ± SD. *n* = 4–5. **P < 0.01 and *P < 0.05, ANOVA with a post hoc test (Tukey’s test).

The representative time course data for the Mt1 iPS cell clones (*n* = 4–5) and the Mt2 iPS cell clones (*n* = 4–5) are shown in Fig. 3a and b, respectively. On the basis of flux analysis among the Mt1 iPS cell clones, the basal respiration, ATP production, and spare respiratory capacity were decreased in the mutation-high clones compared with the mutation-undetectable clones. The basal respiration of Mt1-2^low^, Mt1-3^high^, and Mt1-4^high^ was 91.8%, 72.2%, and 53.8% of that of Mt1-1^low^, respectively. The basal respiration of Mt1-4^high^ was statistically significantly lower than that of Mt1-1^low^ and Mt1-2^low^, and that of Mt1-3^high^ was statistically significantly lower than that of Mt1-1^low^ (Fig. 3c). The ATP production of Mt1-2^low^, Mt1-3^high^, and Mt1-4^high^ was 93.3%, 71.6%, and 28.9% of that of Mt1-1^low^, respectively. The ATP production of Mt1-4^high^ was statistically significantly lower than that of Mt1-1^low^, Mt1-2^low^, and Mt1-3^high^, whereas the ATP production of Mt1-3^high^ was statistically significantly lower than that of Mt1-1^low^ and Mt1-2^low^ (Fig. 3d). The spare respiratory capacity of Mt1-2^low^, Mt1-3^high^, and Mt1-4^high^ was 88.8%, 67.7%, and 25.8% of that of Mt1-1^low^, respectively. The spare respiratory capacity of Mt1-4^high^ was statistically significantly lower than that of Mt1-1^low^ and Mt1-2^low^ (Fig. 3e). The proton leak did not show statistically significant difference (Fig. 3f).

Among the Mt2 iPS cell clones, basal respiration, ATP production, spare respiratory capacity, and proton leak were not different, even in the clone with the highest mtDNA mutation frequency (Mt2-5^high^). The basal respiration of Mt2-8^low^, Mt2-6^high^, and Mt2-5^high^ was 104.4%, 108.4%, and 116.7% of that of Mt2-3^low^, respectively (Fig. 3g). The ATP production of Mt2-8^low^, Mt2-6^high^, and Mt2-5^high^ was 106.3%, 117.3%, and 115.4% of that of Mt2-3^low^, respectively (Fig. 3h). The spare respiratory capacity of Mt2-8^low^, Mt2-6^high^, and Mt2-5^high^ was 107.6%, 85.1%, and 99.9% of that of Mt2-3^low^, respectively (Fig. 3i). Furthermore, the control PSCs (B7, W12, TIG107, and KhES-1) were compared with Mt1-1^low^ and Mt2-3^low^. The basal respiration, ATP production, and spare respiratory capacity of the mutation-undetectable Mt iPS cell clones from both patients were comparable to those of the control PSCs (Supplementary Fig. 3, *n* = 8–9). The analysis was performed independently three times for all sets of cells.

### Dynamics of cytosolic ATP concentration

**Figure 4:**
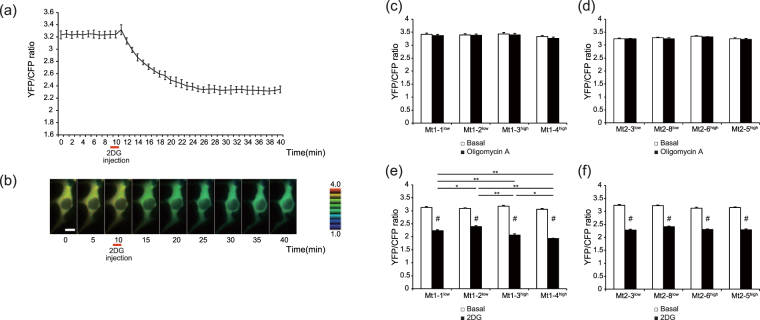
Time-lapse imaging of the Mt iPS cell clones expressing ATeam1.03, a FRET-based ATP biosensor. The time course of the YFP/CFP emission ratio (**a**) and the sequential images (**b**) in a single iPS cell of Mt1-1^low^ expressing ATeam 1.03 were examined after injection of 2DG. 2DG was injected at 10 min. The representative data are expressed as the means ± SD. *n* = 6 (Scale bar, 10 μm). The YFP/CFP emission ratio of the Mt1 iPS cell clones (**c**) and the Mt2 iPS cell clones (**d**) at 10 min (before the oligomycin A injection) and 30 min (20 min after the oligomycin A injection) was measured. The YFP/CFP emission ratio of the Mt1 iPS cell clones (**e**) and the Mt2 iPS cell clones (**f**) at 10 min (before 2DG injection) and 40 min (30 min after 2DG injection) was measured. The data are expressed as the means ± SE. *n* = 10–14 (**c**) (**d**). *n* = 11–16 (**e**) (**f**). **P < 0.01 and *P < 0.05, among the 2DG groups assessed by ANOVA with a post hoc test (Tukey’s test). ^#^*P* < 0.01, compared with basal for each clone assessed using the Student’s *t-*test.

The cytosolic ATP concentration was measured using fluorescence resonance energy transfer (FRET)-based ATP biosensor ATeam 1.03^20^. The A3243G mutation frequencies of the Mt iPS cells with stable genomic integration of ATeam 1.03 (ATeam-iPS cells) were evaluated with the Invader assay^21^; the results showed that the mutation frequencies were not influenced by genomic integration of ATeam 1.03 (Supplementary Fig. 4). The time course and sequential images of the FRET signal (yellow fluorescent protein [YFP]/cyan fluorescent protein [CFP] ratio) are shown in a single Mt1-1^low^ iPS cell expressing ATeam 1.03 after the injection of 2-deoxy-D-glucose (2DG) (Fig. 4a,b). Before the injection of 2DG or oligomycin A (representing the basal ATP concentration), the FRET signals of the Mt1 and Mt2 iPS cell clones were not statistically significantly different. Mitochondrial oxidative phosphorylation (OXPHOS)-dependent and glycolysis-dependent ATP production, respectively, were detected by injecting 2DG or oligomycin A and obtaining sequential FRET signal. The injection of oligomycin A had little effect on the ATP levels between both the Mt1 and Mt2 iPS cell clones (Fig. 4c,d, *n* = 10–14). Among the Mt1 iPS cell clones, OXPHOS-dependent ATP production, analyzed via 2DG, was decreased in the mutation-high clones compared with the mutation-undetectable clones. After the injection of 2DG, FRET signals (YFP/CFP ratio) of 71.5% (Mt1-1^low^), 77.2% (Mt1-2^low^), 65.1% (Mt1-3^high^), and 63.0% (Mt1-4^high^) were observed compared with the basal condition; the FRET signals of Mt1-3^high^ and Mt1-4^high^ were statistically significantly lower than those of Mt1-1^low^ and Mt1-2^low^ (Fig. 4e, *n* = 11–16). Among the Mt2 iPS cell clones, mitochondrial ATP production were not different. FRET signals were observed at 70.6% (Mt2-3^low^), 74.9% (Mt2-8^low^), 73.7% (Mt2-6^high^), and 72.9% (Mt2-5^high^) after the injection of 2DG; there were no statistically significant differences (Fig. 4f, *n* = 11–16). The FRET signals before and after 2DG injection showed statistically significant difference in each clone among the Mt iPS cells (*P < *0.01, Student’s *t-*test) (Fig. 4e,f).

## Discussion

In the present study, the complex I activity, mitochondrial membrane potential, mitochondrial respiration, and mitochondrial ATP production were analyzed among two mutation-undetectable and two mutation-high Mt iPS cell clones from two patients with the A3243G mutation of different pedigrees. In addition, the complex I activity and mitochondrial respiration of the mutation-undetectable Mt iPS cell clones from both patients were compared with those of the control PSCs. The present study shows the following observations: (1) among the Mt1 iPS cell clones, the complex I activity, OCR (contributed to by basal respiration, ATP production, and spare respiratory capacity) and mitochondrial ATP production were decreased in the mutation-high clones compared with the mutation-undetectable clones. Mitochondrial membrane potential after FCCP treatment was decreased in the clone with the highest mtDNA mutation frequency compared with one of the mutation-undetectable clones; (2) among the Mt2 iPS cell clones, the complex I activity was decreased in the clone with the highest mtDNA mutation frequency compared with the other clones, whereas the parameters assessed using FACS, extracellular flux analysis, and ATeam 1.03 showed no differences among any of the clones; (3) the complex I activity and OCR (contributed to by basal respiration, ATP production, and spare respiratory capacity) of the mutation-undetectable Mt iPS cell clones (Mt1-1^low^ and Mt2-3^low^) were comparable to those of the control PSCs. Taken together, the present study suggests that the mitochondrial function of the mutation-undetectable clones is higher than that of the mutation-high clones in Pt1. In addition, the results suggest that the mitochondrial function of the mutation-undetectable clones from both patients is comparable to that of the control iPS cells. This study is the first to our knowledge to analyze the mitochondrial function, via a variety of assays, of iPS cells derived from two genetically unrelated patients with the A3243G mutation.

Mitochondria generate ATP via OXPHOS, which is driven by the electron transport chain (ETC)^22^. The ETC consists of five sequential multiprotein complexes, I (NADH ubiquinone reductase), II (succinate ubiquinone reductase), III (ubiquinol cytochrome C reductase), IV (cytochrome oxidase), and V (ATP synthase); oxygen is reduced to water by complex IV, and ATP is finally produced by complex V^22^. Complexes I, III, IV, and V are composed of subunits encoded in both mtDNA and the nuclear genome, and complex II consists of subunits encoded only in the nuclear genome^22^. A3243G is a point mutation in the mitochondrial tRNA^Leu(UUR)^ gene that causes a respiratory defect chiefly due to a deficiency of complex I. Regarding the mechanism of complex I deficiency in patients with the A3243G mutation, abnormal tRNA conformation^23^, lack of 3′-end processing, CCA addition^24^, aminoacylation^25,26^, transcription termination^27^, and/or 5-taurinomethyluridine modification^28,29^ are considered to cause insufficient translation of leucine. Our findings on decreased complex I activity in mutation-high iPS cell clones are consistent with the results of previous reports^23–29^.

In Pt2, complex I activity was decreased in Mt2-5^high^ (mutation frequency: 85%), but not in Mt2-6^high^ (mutation frequency: 70%). We speculate a threshold effect of mitochondrial heteroplasmy, which was reported using the cytoplasmic hybrid, or cybrid, method^30^. In typical cases of pathogenic mitochondrial tRNA mutations, when the proportion of mutant mtDNA is lower than 85% to 90%, the cell maintains its normal physiological function and phenotype^30^. After the ratio of mutant mtDNA exceeds the threshold value, the energetic biological capacity, such as complex I activity in this case, is impaired^30^. Our results suggest that the threshold value to maintain complex I activity is between 70% and 85% among the Mt2 iPS cells.

In Mt2-5^high^, the mitochondrial membrane potential after treatment with FCCP, mitochondrial respiration and mitochondrial ATP production were not different from those of the other Mt2 iPS cell clones, though the complex I activity was decreased. We speculate that a common molecular mechanism, which depends on the A3243G mutation, can decrease the mitochondrial function assessed in the present study. Therefore, a compensatory mechanism could maintain the normal functioning of mitochondrial OXPHOS in Mt2-5^high^. In previous studies, some mtDNA mutations, including position 3243 mutations, have been found to be accompanied by downregulation of complex I but upregulation of complexes II, III, and IV^13,31,32^. The counter-regulation of complexes II–IV may contribute to the ability of Mt2-5^high^ to maintain normal mitochondrial function. Regarding Pt1, although the compensatory mechanism supporting normal mitochondrial function should work in the mutation-high iPS cell clones, the A3243G mutation strongly decreased mitochondrial function beyond the capacity of the compensatory mechanism. The difference in mitochondrial function between the Mt1 and Mt2 iPS cells may be explained by the unique nuclear genome information of each patient; that is, the genome background of each patient may contribute to the compensatory capacity to a certain degree. It is highly likely that the mitochondrial function of the Mt iPS cells is dependent on their genomic background, in addition to heteroplasmy and the threshold effect.

The mutation-undetectable iPS cell clones should be very important for cell replacement therapy if their mitochondrial function is comparable to iPS cells from healthy subjects. For this reason, the mitochondrial function of the mutation-undetectable Mt iPS cell clones (Mt1-1^low^ and Mt2-3^low^) was compared with three control iPS cell lines and one ES cell line (Supplementary Fig. 1, 3). Complex I activity and mitochondrial respiration were assessed, and the results showed that the complex I activity and OCR (contributed to by basal respiration, ATP production, and spare respiratory capacity) were comparable between the mutation-undetectable Mt iPS cell clones and the control PSCs. These results suggest that the mitochondrial function of the mutation-undetectable Mt iPS cell clones is comparable to the control iPS cells.

A recent study on mitochondria in PSCs reported that these cells contain immature and inactive mitochondria, while still possessing functional respiratory complexes, and rely heavily on anaerobic glycolysis compared with somatic cells^33–36^. In the present study, the ATP production of OXPHOS (Fig. 4e,f) was much lower than that of glycolysis (Fig. 4c,d) in all Mt iPS cell clones, and these results are consistent with previous studies^33–36^. According to previous reports^33–36^, identification of mitochondrial functional defects could be more difficult in iPS cells with mitochondrial mutations than in somatic cells because the mitochondrial functions of the mutation-high and the mutation-undetectable clones may not be significantly different in the stem cell state. The present study demonstrates that the four methods used in this study could be useful to assess differences in mitochondrial function caused by the A3243G mutation in iPS cells.

Patients with mitochondrial A3243G mutations present with various types of clinical phenotypes. In the current study, the clinical features of Pt1 and Pt2 were markedly different: the former showed only diabetes, while the latter showed diabetes and MELAS. We speculate that the mutation frequency in neurons and cardiomyocytes might be much higher in Pt2 than in Pt1. Regarding the diabetic phenotype, both patients had insulin-dependent diabetes mellitus with a similar severity of insufficient insulin secretion in their thirties. In terms of diabetes, the following factors, which are involved in the pathological mechanisms of the disease, should be carefully considered. (1) Heteroplasmy: organ function in mitochondrial diseases strongly relies on mitochondrial heteroplasmy in the somatic cells of the affected tissues. Although the fibroblasts obtained from skin biopsies in the two patients showed comparable mutation frequencies (18%: Pt1, 24%: Pt2), the heteroplasmy of beta cells may be different. The degree of heteroplasmy is unknown in both patients, because clinical limitations make sampling of beta cells difficult. (2) Lifestyle factors: dietary habits, exercise, and various environmental-related factors strongly affect the clinical course of diabetes. (3) Nuclear genome background: genetic factors, in addition to mtDNA mutation, also affect the onset or progression of diabetes. Therefore, the clinical aspects of diabetes should be carefully considered in both patients.

It is crucial for regenerative medicine to clarify whether iPS cells have the potential to differentiate into tissue-specific cells. We have previously reported the capability of ES cells and iPS cells to differentiate into vascular endothelial cells^37–40^, adipocytes^17,18,41^, steroid-producing cells^42^, and chondrocytes^43^. In patients with mitochondrial diabetes, beta cells cannot satisfy the energetic or signaling requirements for glucose-stimulated insulin secretion, owing to impaired ETC function in the mitochondria^44^. Thus, pancreatic beta cells generated from PSCs possessing normal mitochondrial function are an essential resource for cell replacement therapy for mitochondrial diabetes. Recently, two different research groups have differentiated human PSCs into insulin-containing cells expressing beta cell transcription factors; in addition, glucose-stimulated insulin secretion *in vitro* and reversal of hyperglycemia in diabetic mice after transplantation have been reported^45,46^.

Several studies have also reported that mutation-undetectable iPS cells are generated from patients with the A3243G mutation, as demonstrated in our previous study^12,13,16^. Given these previous reports, it is highly probable that mutation-undetectable iPS cells can be generated from the fibroblasts of any patients with the mitochondrial A3243G mutation. The present study of iPS cells from two patients suggests that the mitochondrial function of the mutation-undetectable iPS cell clones is comparable to that of the control iPS cells. The results also suggest that the same phenomenon could be present in mutation-undetectable iPS cells established from almost all patients with the A3243G mutation. This phenomenon highlights the potential of this approach in the future development of autologous cell replacement therapy for patients with the mitochondrial A3243G mutation.

In summary, we established isogenic iPS cells with undetectable levels or high levels of mtDNA mutation from two diabetic patients with the A3243G mutation. The present study suggests that the mitochondrial function of the mutation-undetectable iPS cell clones is comparable to that of the control iPS cells. After the development of appropriate and safe methods for the differentiation of iPS cells into beta cells in the future, our approach to generating mutation-undetectable iPS cells holds promise in the development of autologous cell replacement therapy for patients with mitochondrial diabetes due to the A3243G mutation.

## Materials and Methods

### Patient information

Pt1 was a 38-year-old male with a family history of MELAS. His mother had diabetes mellitus, cardiomyopathy, and hearing loss. He was hospitalized because of severe weight loss, hydrodipsia, and polyuria when he was 30 years old. He was given a diagnosis of insulin-dependent diabetes mellitus and treated with insulin therapy. Pt2 was a 46-year-old female who had diabetes mellitus, hearing loss, epilepsy, and cardiomyopathy. She was diagnosed with gestational diabetes mellitus during her first pregnancy, when she was 24 years old. She was treated with insulin therapy for diabetic ketoacidosis when she was 31 years old. She was hospitalized repeatedly because of epilepsy and acute exacerbation of chronic heart failure.

### iPS cell clones

Several iPS cell lines were generated from Pt1 (Mt1 iPS cells) and Pt2 (Mt2 iPS cells) using a retrovirus encoding *OCT4*, *SOX2, c-MYC*, and *KLF4*. These iPS cell clones had normal karyotypes and met pluripotency criteria^12^. The A3243G mutation frequencies of the Mt iPS cell clones were evaluated by using the Invader assay^21^. An analysis of the same clones at various passage numbers revealed that no induction of the mutation occurred in the mutation-undetectable Mt iPS cell clones and that the mutation frequencies of the mutation-high Mt iPS cell clones were relatively constant across passages^12^. Moreover, the mtDNA content of the Mt iPS cells after passage 20 was similar to that of human ES cells^12^. The following eight clones from the Mt1 and Mt2 iPS cells were selected and used at passage 20–40 in further analyses: two mutation-undetectable clones (Mt1-1^low^, Mt1-2^low^) and two mutation-high clones (mtA3243G mutation frequencies of 77% [Mt1-3^high^] and 91% [Mt1-4^high^]) from the Mt1 iPS cells; and two mutation-undetectable clones (Mt2-3^low^, Mt2-8^low^), which were randomly selected from among six mutation-undetectable clones, and two mutation-high clones (mtA3243G mutation frequencies of 70% [Mt2-6^high^] and 85% [Mt2-5^high^]), which were selected because they possessed the lowest and highest mutation frequencies, respectively, among the four mutation-high clones, from the Mt2 iPS cells. The clone number of the Mt iPS cells was based on a previous report^12^. In addition, three iPS cell lines (B7, W12, and TIG107) and one ES cell line (KhES-1) were used as controls.

### Cell culture

Mitochondrial diabetes-specific iPS cells were cultured on mitomycin C (Sigma-Aldrich, St. Louis, MO, USA)-treated mouse embryonic fibroblast cell line (SNL) cells^47^ by using primate ES cell medium (ReproCELL, Yokohama, Japan) supplemented with 4 ng/ml of basic fibroblast growth factor (Wako, Osaka, Japan). The culture medium was changed every day. The iPS cells were passaged every 5–7 days after removal of the feeder cells using 0.25% trypsin (Nacalai Tesque, Kyoto, Japan) with 0.1 mg/ml collagenase type IV (Thermo Fisher Scientific, Waltham, MA, USA) and 20% KnockOut Serum Replacement (Thermo Fisher Scientific). After removal of the mouse feeder cells, the iPS cells were incubated in Accumax (Funakoshi, Tokyo, Japan) for 10 min and dissociated into single cells. Single iPS cells were seeded on growth-factor-reduced Matrigel (#356231; Corning, New York, NY, USA)-coated dishes using mTeSR1 medium (STEMCELL Technologies, Vancouver, Canada) supplemented with 10 μM Y-273652 (Nacalai Tesque).

### Measurement of complex I activity

The activity of complex I was measured using a Complex I Enzyme Activity Microplate Assay Kit (ab109721; Abcam, Cambridge, MA, USA) according to the manufacturer’s protocol. Briefly, protein was extracted from iPS cells using the detergent provided by the manufacturer. The protein concentration was measured using DC Protein Assay Reagent (BIO-RAD, Hercules, CA, USA), and all samples were diluted to the manufacturer-recommended concentration. Equal amounts of each sample were loaded onto the plate and incubated for 3 hours at room temperature. After incubation, NADH and dye provided by the manufacturer were loaded onto the plate, and the absorbance at 450 nm in each well was measured at 1-min intervals for 30 min. The linear rate of increase in absorbance at 450 nm was calculated.

### FACS analysis

FACS analysis was performed to evaluate the significance of the A3243G mutation by using two types of mitochondria-specific labels. MitoTracker Green (Thermo Fisher Scientific) and TMRE (Thermo Fisher Scientific), respectively, to distinguish total mitochondrial mass and respiration. The iPS cells were incubated with 100 nM MitoTracker Green and 50 nM TMRE at 37 °C for 15 min. The iPS cells were dissociated into single cells as described above, collected in Hank’s balanced salt solution (HBSS) medium (Thermo Fisher Scientific) supplemented with 2% FBS (Sigma-Aldrich), and analyzed using flow cytometry (LSRFortessa Cell Analyzer; BD Biosciences, San Jose, CA, USA). Next, FCCP (Sigma-Aldrich), a respiratory uncoupler, was added to a final concentration of 4 μM. Data were analyzed using FlowJo software (Treestar, Ashland, OR, USA).

### Extracellular flux analysis

The OCR was measured using an XF96 Extracellular Flux Analyzer (Seahorse Bioscience, Billerica, MA, USA) according to the manufacturer’s protocol. The iPS cells were dissociated into single cells as described above, and 4 × 10^4^ cells were seeded onto an XF96 cell-culture microplate coated with Matrigel using mTeSR1 medium containing 10 μM compound Y-27632. The next day, the medium was replaced with XF assay medium^48^, followed by sequential injection of 2 μM oligomycin A, 62.5 nM FCCP, 10 mM antimycin A (Sigma-Aldrich), and 10 mM rotenone (Sigma-Aldrich). The results were normalized to the total protein content in each well, which was determined using DC Protein Assay Reagent.

### Generation of a stable iPS cell line (ATeam-iPS cells)

The use of the previously developed ATeam 1.03 allowed for direct monitoring of cytosolic ATP levels in a single living cell^20^. Briefly, genetically encoded ATP biosensor ATeam 1.03 is based on the principle of FRET and is specific for ATP. It is composed of the epsilon subunit of *Bacillus subtilis* F_o_F_1_-ATP synthase, a variant of CFP, and a variant of YFP. Large conformational changes of the subunit induced by ATP binding lead to an increase in the FRET signals (YFP/CFP ratio). To evaluate the ATP levels and dynamics of the Mt iPS cells, ATeam-iPS cells were generated via stable genomic integration of ATeam 1.03. ATeam 1.03 was cloned via the piggyBac vector by using an In-Fusion HD Cloning Kit (Takara, Kusatsu, Japan). The iPS cells on Matrigel-coated dishes were transfected with FuGENE HD (Promega, Madison, WI, USA) according to the manufacturer’s protocol. Drug selection was performed using 0.5 μg/ml puromycin (Sigma-Aldrich). The ATeam-iPS cells were cultured on puromycin-resistant SNL feeder cells generated using the pMXs-IP vector.

### Imaging and data analysis

Stable iPS cell lines were dissociated into single cells and plated on a glass-bottom dish (MatTek Corporation, Ashland, MA, USA). The iPS cells were washed with PBS and incubated in HBSS-based medium for 15 min. The HBSS medium was prepared according to the manufacturer’s instructions (Thermo Fisher Scientific), and 20 mM HEPES (Nacalai Tesque), 1 mM sodium pyruvate (Sigma-Aldrich), and 2 mM GlutaMax-1 (Thermo Fisher Scientific) were added. Ten minutes after monitoring began, 2DG (Sigma-Aldrich) or oligomycin A (Sigma-Aldrich) was injected manually. The final concentrations were 20 mM and 2 μM, respectively. Imaging analysis of single iPS cells was performed on a Nikon Ti-E-PFS inverted microscope (Nikon, Tokyo, Japan) by using a dry objective (CFI Plan Apo λ 40 × NA 0.95; Nikon) and the following filter sets (Semrock, Rochester, NY, USA) for dual-emission-ratio imaging of ATeam 1.03: 438/24, DM458, and 483/32 (CFP) and 542/27 (YFP). An ORCA-AG cooled charge-coupled device camera (Zyla4.2 sCMOS, Andor Technology, Belfast, UK) was used to capture fluorescence images. The microscope system was operated by NIS-Elements (Nikon). For the data analysis, regions of interest (ROIs) were selected in the cytosol. The YFP/CFP intensity ratio of the ROIs was calculated at every time point using MetaMorph (Molecular Devices, Sunnyvale, CA, USA).

### Statistics

The results are expressed as the means ± SD or SE. Unless otherwise indicated, the statistical significance of the differences in mean values among the four clones for each patient were assessed by analysis of variance (ANOVA) with a post hoc test (Tukey’s test) using Statcel2 Software (OMS Publishing, Saitama, Japan). *P*-values less than 0.05 were considered to indicate statistical significance and are indicated by asterisks in the figures.

### Study approval

The studies presented here were approved by the Ethics Committees of Kyoto University and all methods were performed in accordance with the relevant guidelines and regulations. All patients provided written informed consent.

## Electronic supplementary material

Supplementary |Information
