# Supplementary material for: Analysis of mitochondrial function in human induced pluripotent stem cells from patients with mitochondrial diabetes due to the A3243G mutation
Source: Sci Rep. 2018 Jan 17;8:949. doi: 10.1038/s41598-018-19264-7 (PMC5772054; doi:10.1038/s41598-018-19264-7)
Supplement: Supplementary file 1 — Supplementary |Information [file 41598_2018_19264_MOESM1_ESM.pdf]

Analysis of mitochondrial function in human induced pluripotent stem cells from patients with mitochondrial diabetes due to the A3243G mutation

Masaki Matsubara[1,2], Hajime Kanda[1], Hiromi Imamura[3], Mayumi Inoue[1,2], Michio Noguchi[1], Kiminori Hosoda[4], Akira Kakizuka[3], and Kazuwa Nakao[1, *]

1 Medical Innovation Center, Kyoto University Graduate School of Medicine, 53 Shogoin Kawahara-cho, Sakyo-ku, Kyoto 606-8507, Japan.

2 Department of Diabetes, Endocrinology and Nutrition, Kyoto University Graduate School of Medicine, 54 Shogoin Kawahara-cho, Sakyo-ku, Kyoto 606-8507, Japan.

3 Kyoto University Graduate School of Biostudies, Yoshida-Konoecho, Sakyo-ku, Kyoto 606-8501, Japan.

4 Department of Human Health Science, Kyoto University Graduate School of Medicine, 53 Shogoin Kawahara-cho, Sakyo-ku, Kyoto 606-8507, Japan.

Corresponding author: Kazuwa Nakao, Medical Innovation Center, Kyoto University Graduate School of Medicine, 53 Shogoin Kawahara-cho, Sakyo-ku, Kyoto 606-8507, Japan.

Phone: +81-75-366-7450, FAX: +81-75-752-7140, E-mail address: nakao@kuhp.kyoto-u.ac.jp

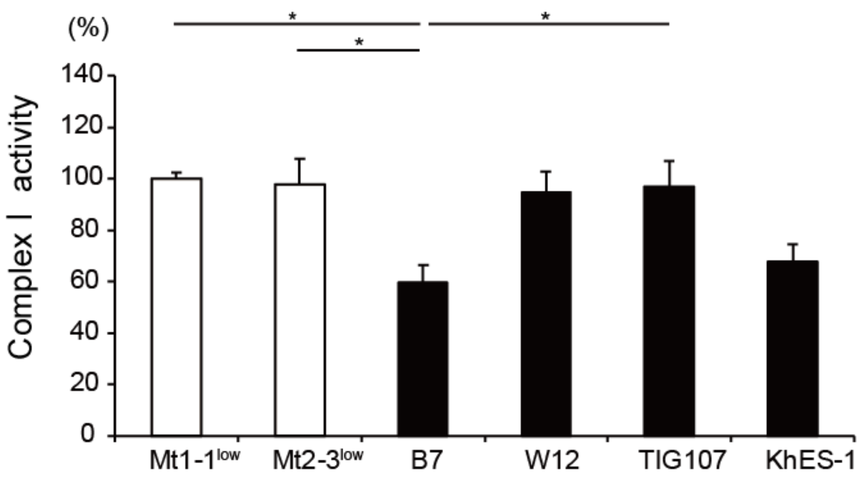

Supplementary Figure 1

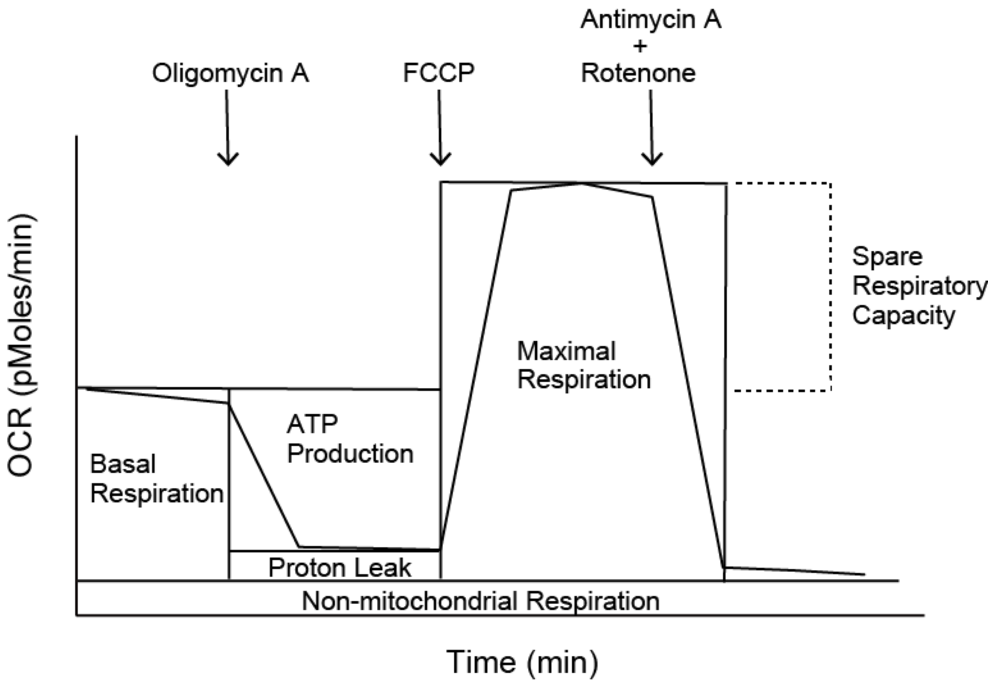

Supplementary Figure 2

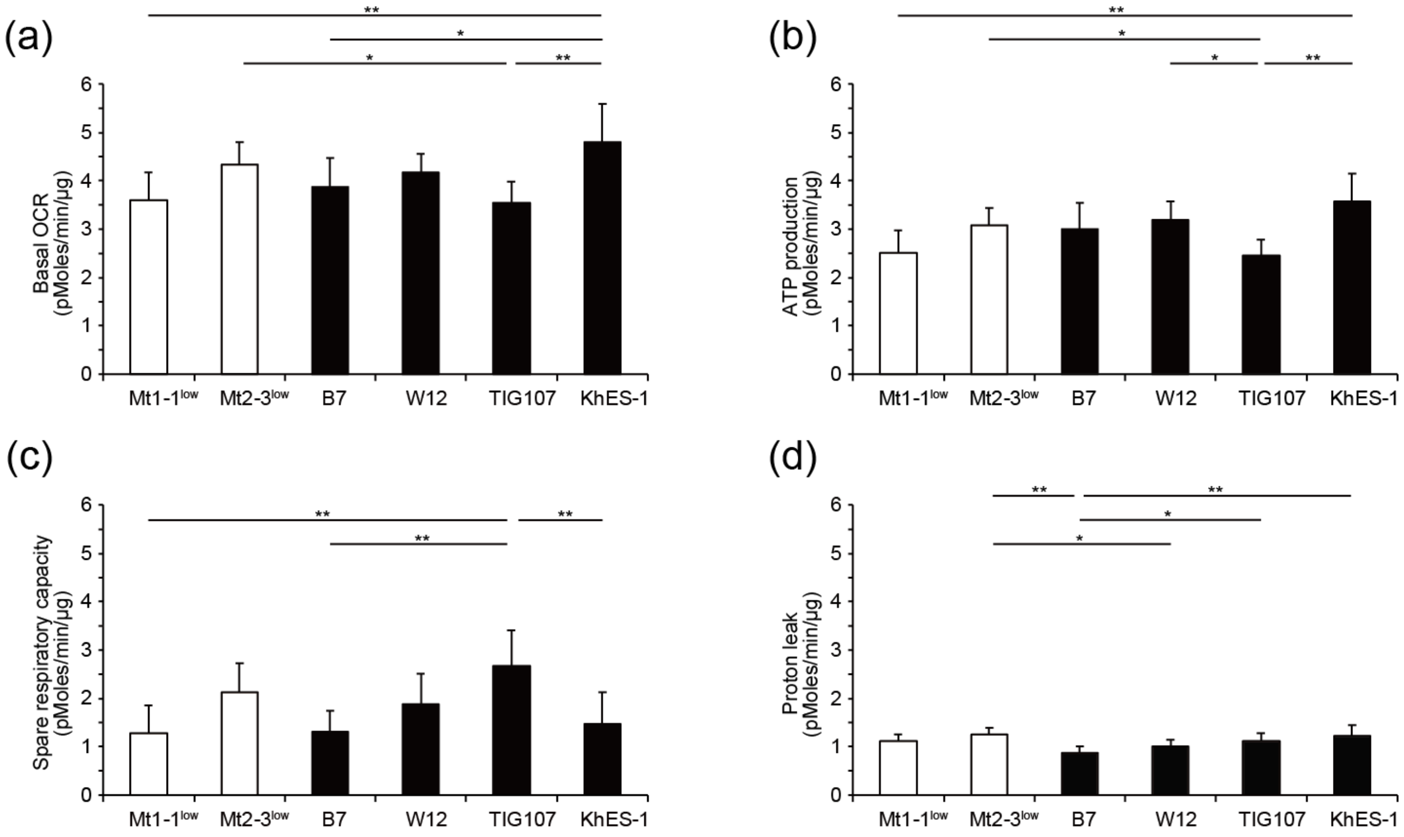

Supplementary Figure 3

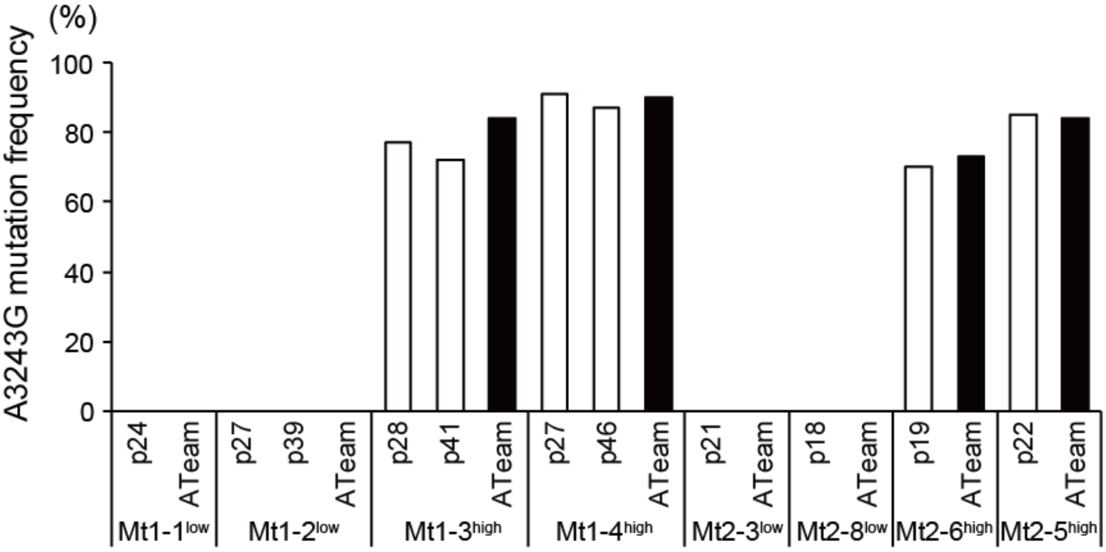

Supplementary Figure 4

Figure Legends

Supplementary Figure 1. Complex I activity of the mutation-undetectable Mt iPS cell clones (Mt1-1^{low} and Mt2-3^{low}) and the control pluripotent stem cells (PSCs).

The data were normalized to that of Mt1-1^{low}. The complex I activity of Mt1-1^{low} and Mt2-3^{low} was comparable to that of the control PSCs (B7, W12, TIG107 and KhES-1). The data are expressed as the means ± SE. n=10-15. *P<0.05, ANOVA with a post hoc test (Tukey's test).

Supplementary Figure 2. Assessment of mitochondrial activity by measuring the oxygen consumption rate (OCR) using an XF96 Extracellular Flux Analyzer.

Mitochondrial activity was evaluated via the serial addition of oligomycin A, FCCP and antimycin A, plus rotenone.

Supplementary Figure 3. The oxygen consumption rate (OCR) of the mutation-undetectable Mt iPS cell clones (Mt1-1^{low} and Mt2-3^{low}) and the control pluripotent stem cells (PSCs) was measured using an XF96 Extracellular Flux Analyzer.

All parameters: basal OCR (a), ATP production (b), spare respiratory capacity (c) and proton leak (d) of the mutation-undetectable Mt iPS cell clones were comparable to those of the control PSCs (B7, W12, TIG107 and KhES-1). The data are expressed as the means ± SD. n=8-9. **P<0.01 and *P<0.05, ANOVA with a post hoc test (Tukey's test).

Supplementary Figure 4. A3243G mutation frequencies in the Mt iPS cell clones.

A3243G mutation frequencies in the Mt iPS cell clones at the indicated culture passage (p) numbers and with stable genomic integration of ATeam1.03 (ATeam) are shown.
